# Nissl Granules, Axonal Regeneration, and Regenerative Therapeutics: A Comprehensive Review

**DOI:** 10.7759/cureus.47872

**Published:** 2023-10-28

**Authors:** Manya Bhati, Swedaj Thakre, Ashish Anjankar

**Affiliations:** 1 Medicine, Jawaharlal Nehru Medical College, Datta Meghe Institute of Higher Education and Research, Wardha, IND; 2 Biochemistry, Jawaharlal Nehru Medical College, Datta Meghe Institute of Higher Education and Research, Wardha, IND

**Keywords:** specialty treatment, cellular biology, human physiology, treatment choices, adult neurology

## Abstract

Nissl granules, traditionally recognized for their pivotal role in protein synthesis within neuronal cell bodies, are emerging as intriguing components with far-reaching implications in the realm of regenerative therapeutics. This abstract encapsulates the essence of a comprehensive review, exploring the nexus between Nissl granules, axonal regeneration, and their transformative applications in regenerative medicine. The molecular intricacies of Nissl granules form the foundation of this exploration, unraveling their dynamic role in orchestrating cellular responses, particularly in the context of axonal regeneration. As we delve into the interplay between Nissl granules and regenerative processes, this review highlights the diverse mechanisms through which these granules contribute to neuronal repair and recovery. Beyond their conventional association with neurobiology, recent advancements underscore the translational potential of Nissl granules as therapeutic agents. Insights into their involvement in enhancing axonal regeneration prompt a reconsideration of these granules as key players in the broader field of regenerative medicine. The abstract encapsulates evidence suggesting that modulating Nissl granule-related pathways holds promise for augmenting tissue regeneration, extending their applicability beyond the confines of the nervous system. This review aims to serve as a valuable resource for medical professionals, researchers, and clinicians seeking to comprehend the multifaceted role of Nissl granules in regenerative therapeutics. By illuminating the intricate connections between Nissl granules, axonal regeneration, and therapeutic applications, this work aspires to catalyze further research and innovation, ultimately contributing to the evolution of regenerative strategies that harness the innate reparative capacities within cellular constituents.

## Introduction and background

In the ever-evolving landscape of regenerative medicine, the exploration of cellular constituents takes center stage as we strive to unlock the full potential of therapeutic interventions. Nissl granules, initially recognized for their role in neuronal protein synthesis, have recently garnered attention for their intriguing applications in regenerative therapeutics. Nissl granules are not directly involved in therapeutic manipulation as they are an intrinsic component of the neuron and manipulating them directly is not a common therapeutic approach. As medical professionals, our pursuit of innovative approaches to enhance tissue repair and regeneration compels us to delve into the nuanced world of Nissl granules and their promising implications for regenerative medicine [1-4]. Nissl granules, residing predominantly in the cell bodies of neurons, have long been recognized as vital hubs for protein synthesis. Traditionally studied in the context of neurobiology, recent research has expanded our understanding of these granules beyond their role in normal neuronal function. This review endeavors to explore the emerging evidence that positions Nissl granules as not only integral to neuronal health but also as potential mediators of regenerative processes in various tissues [5].

As we embark on this exploration, we will navigate the molecular intricacies of Nissl granules, unraveling their role in orchestrating cellular responses to injury and stress. Furthermore, we will examine the translational potential of harnessing Nissl granule-related mechanisms for therapeutic purposes, shedding light on their applications in tissue regeneration, repair, and recovery [6-9]. In synthesizing current knowledge and delving into the intersection of neurobiology and regenerative medicine, this review seeks to provide a comprehensive overview for medical professionals, researchers, and practitioners alike. By understanding the versatile applications of Nissl granules, we aim to contribute to the ongoing dialogue surrounding regenerative therapeutics, fostering a new era where the innate capabilities of cellular constituents are harnessed to redefine the landscape of patient care and healing [10].

## Review

Search methodology

**Figure 1:**
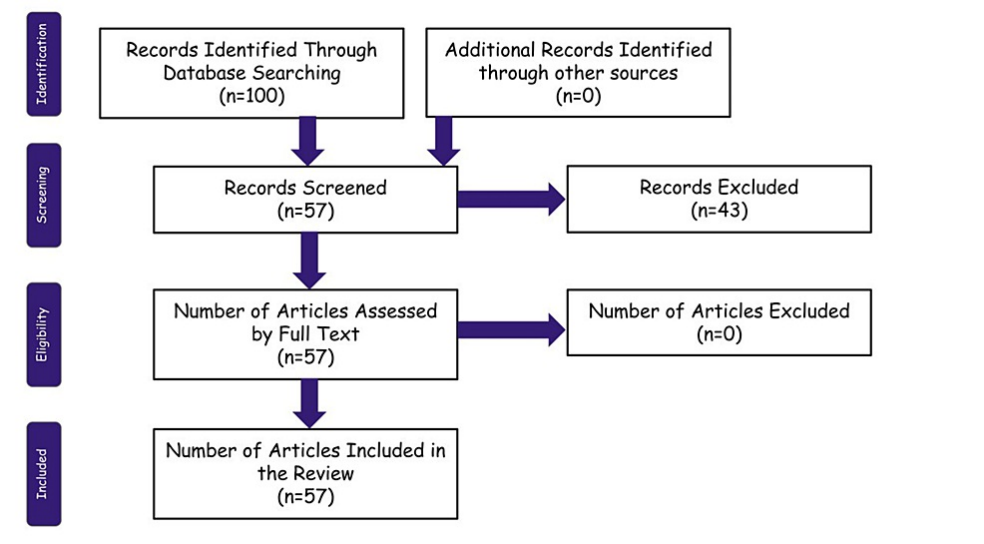
Preferred Reporting Items for Systematic Reviews and Meta-Analyses (PRISMA) diagram of the review process The figures are the author's creation.

This review article was made after careful assessment and evaluation of the various research studies conducted across the globe on the primary functions of Nissl granules and axonal regeneration and their involvement in regenerative medicine and therapeutics. The primary database used for the search is PubMed, along with certain research studies obtained from other databases and sources. Specific keywords involved Nissl granules, regenerative therapeutics, axonal regeneration, neuronal structures, and neurodegenerative disorders. The duration of the publication of articles considered for the purpose of review is within the past 75 years. Those articles that could not put forth a definite and precise conclusion and those whose results were found to be doubtful are excluded. A Preferred Reporting Items for Systematic Reviews and Meta-Analyses (PRISMA) flow diagram of the review indicating the screening process is summarized in Figure 1.

Basic physiology behind Nissl granules and axonal regeneration

Comprehending the intricacies of Nissl granules and their implications in axonal regeneration is fundamental for medical professionals engaged in exploring regenerative therapeutics. Nissl granules, initially identified by Franz Nissl as aggregations of rough endoplasmic reticulum in neuronal cell bodies, are integral to the physiological machinery governing protein synthesis within neurons. The basic physiology of Nissl granules involves their crucial role in synthesizing proteins vital for neuronal structure and function. These granules are enriched with ribosomes and endoplasmic reticulum, forming a distinctive cytoplasmic inclusion in the neuronal soma. Traditionally viewed through the lens of neurobiology, recent research has expanded our understanding to recognize Nissl granules as sites of protein synthesis and as dynamic entities responsive to cellular stress and injury. Utilization of therapeutic strategies that involve direct manipulation of these entities can open doors for newer possibilities in the field of neurodegenerative disorders. Specific drug targets like amyloid-β (Aβ) and tau proteins, glutamate receptors, cholinergic system, and oxidative stress pathways could be triggered by direct or indirect manipulation of Nissl granules [11].

**Figure 2:**
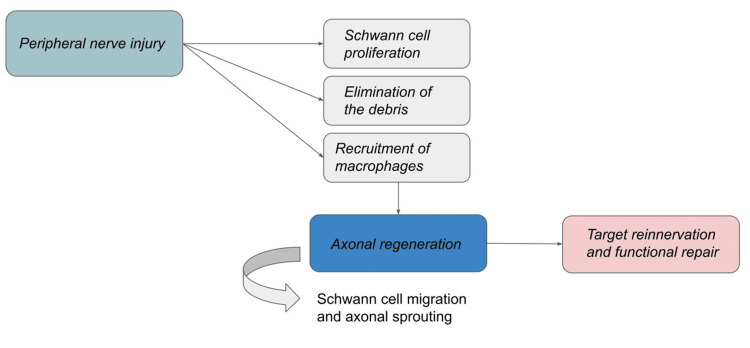
Schematic representation of axonal regeneration The figures are the author's creation.

Our understanding of peripheral nerve regeneration has improved, thanks to rodent models of nerve damage, but therapeutic applications have been limited, in part because these models do not accurately reflect the condition in humans. Axons are frequently required to travel significantly greater distances in human injuries than in mice, and injury causes distal nerve fibers and target tissues to remain without axonal contact for a protracted period of time. Lack of touch with proximal neurons causes distal Schwann cells to atrophy, which inhibits axonal extension by reducing the expression of neurotrophic growth factors, altering the extracellular matrix, and removing the basal lamina of the Schwann cell. Understanding the orchestration of these granules in the aftermath of neural injury is pivotal for unlocking strategies to enhance regenerative processes. In conclusion, a comprehensive review of Nissl granules, axonal regeneration, and their applications in regenerative therapeutics is poised at the intersection of neurobiology and regenerative medicine. This understanding holds promise for advancing therapeutic approaches that capitalize on the inherent reparative mechanisms within neuronal constituents, paving the way for innovative strategies in the realm of regenerative medicine [12-16]. Figure 2 shows the schematic diagram of axonal regeneration.

Neuronal growth and plasticity

Our understanding of neuroscience is based on neuronal plasticity, which is frequently referred to as the brain's extraordinary adaptability. This concept has important clinical consequences. The ability of the brain to rearrange its anatomical and functional organization in response to a variety of inputs, experiences, and environmental changes is known as this phenomenon. Recognizing neural plasticity's dynamic nature is essential for medical professionals because it is essential for learning, memory, and damage recovery. While functional plasticity includes adjustments to synaptic strength and the rewiring of brain networks, structural plasticity includes the development of new synapses, changes in dendritic branching, and even neurogenesis. This innate ability to adapt persists into adulthood and goes beyond developmental phases. Clinical rehabilitation plans for individuals recuperating from neurological diseases like strokes or traumatic brain injuries must take into account neural plasticity. Additionally, knowledge of plasticity mechanisms aids in the creation of therapeutic treatments for mental illnesses and neurodegenerative diseases. A foundation for improving patient care and neurological research is laid by embracing the idea of neural plasticity, which emphasizes the dynamic nature of the brain and the potential for recovery, adaptation, and development [17-25].

**Figure 3:**
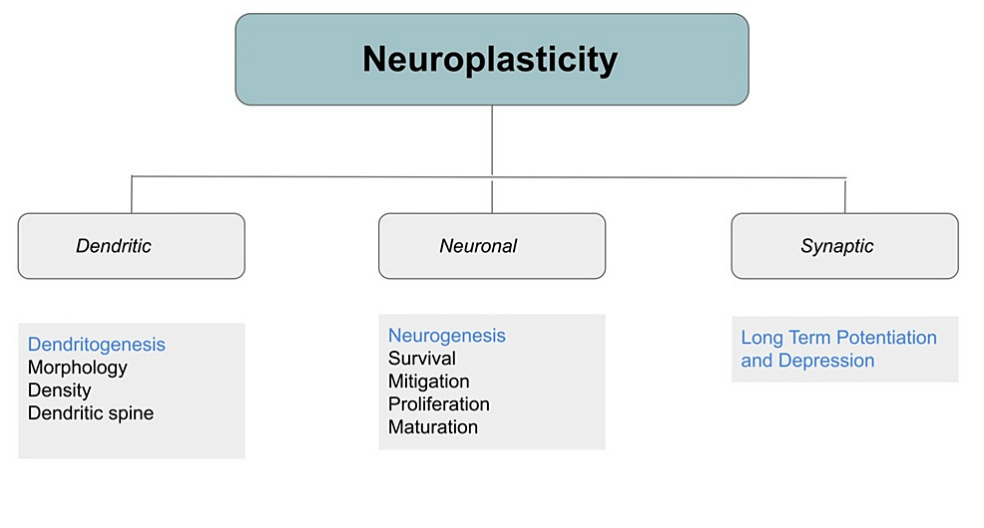
Flowchart showing neuroplasticity The figures are the author's creation.

Nissl granules are involved in protein synthesis, which is essential for neuronal growth and synaptic plasticity. In the context of regenerative medicine, promoting the synthesis of proteins within Nissl granules using neuroprotective strategies can aid in guiding axonal growth and facilitating connections with target cells during nerve regeneration. Manipulating Nissl granule formation and protein synthesis pathways could lead to more effective strategies for neuronal regrowth and re-establishing functional neural circuits. Neuronal plasticity and Nissl granules are interconnected in the functioning and adaptability of neurons within the nervous system. Neuronal plasticity refers to the brain's ability to change and reorganize its neural connections in response to experience, learning, and environmental stimuli. Nissl granules, on the other hand, are involved in protein synthesis. The association between neuronal plasticity and Nissl granules lies in the crucial role that protein synthesis plays in shaping and supporting plasticity processes [26-28]. Figure 3 shows the types of neuroplasticity.

The association of neuronal plasticity and Nissl granules lies in the fundamental role of protein synthesis in shaping and supporting plasticity processes. The proteins synthesized within Nissl granules are crucial for synaptic plasticity, dendritic spine remodeling, axonal growth, and other forms of neuronal adaptation. Understanding this connection helps researchers elucidate the molecular mechanisms underlying neural plasticity and how it contributes to learning, memory, and adaptive behaviors in the brain [29-32].

Disease mechanisms and therapeutic targets

Atypical Nissl granule distribution and protein production problems in neurons are linked to a number of neurological diseases, including amyotrophic lateral sclerosis (ALS) and Alzheimer's disease. Finding possible therapeutic targets for these severe illnesses may be aided by research into the underlying processes of these disturbances. Nissl granule-associated pathways are the focus of regenerative medicine techniques that may offer novel ways to stop the progression of such neurological illnesses. In several neurological conditions, abnormal Nissl granule distribution is seen, and these aberrations can have a big impact on the survival and function of neurons. Although Nissl granules themselves are not directly the target of therapeutic intervention, recognizing their relationship to particular disorders can be very useful in planning a therapeutic path [33].

Ethical considerations in regenerative therapies are of paramount importance to the medical community. These cutting-edge therapies, which include tissue engineering, gene editing, and stem cell therapies, have enormous promise to benefit patients, but they also create difficult ethical and practical issues. The main ethical considerations are the protection of patients, informed consent, fair access, and the distinction between experimental interventions and therapies. As medical professionals, our responsibility is to uphold the highest standards of ethical practice, safeguarding patient rights and welfare while advancing the boundaries of regenerative medicine within a rigorous and ethically sound framework. Here are some examples of diseases associated with abnormal Nissl granule distribution and potential therapeutic treatments.

Alzheimer's Disease

In the context of Alzheimer's disease, regenerative treatments entail using the body's built-in repair mechanisms to encourage neuronal regeneration and functional recovery. Recent developments in the study of neurogenesis and stem cell research have laid the groundwork for investigating regenerative therapies. With their capacity to change into diverse cell types, including neurons, stem cells present possible methods for regenerating injured or missing brain tissue. Additionally, encouraging endogenous neurogenesis - the development of new neurons in the brain - has come to be recognized as a potential approach. Manipulation of Nissl granules to trigger specific targets like Aβ and tau proteins is one of the suggested ways to prevent the disease progression. However, a multimodal strategy is necessary due to the complexity of Alzheimer's pathogenesis. Inflammation, oxidative stress, and the complex web of signaling pathways implicated in neurodegeneration should all be addressed by regenerative treatments, in addition to their primary focus on cellular replacement. Individualized regenerative techniques suited to each patient may be guided by precision medicine, which incorporates unique genetic and molecular profiles.

Although regenerative therapies for Alzheimer's disease are still in their nascent stages, the changing environment offers hope for paradigm-shifting treatments. To take these discoveries from the lab to the patient's bedside, neuroscientists, doctors, and specialists in regenerative medicine must work together. To provide meaningful and disease-modifying interventions for this difficult condition, medical professionals must actively participate in research, clinical trials, and the ethical application of regenerative approaches [34-37].

Amyotrophic Lateral Sclerosis

The selective degradation of motor neurons, which causes muscle atrophy, weakening, and eventually paralysis, is the defining pathology of ALS. Regenerative therapy aims to address the underlying cause of ALS by encouraging the regeneration of damaged neurons or replacing lost cells, whereas current treatments concentrate on supportive care. Because of their capacity to develop into motor neurons and potentially refill the depleted neuronal pool, stem cell therapies in particular have drawn attention [38].

Additionally, cutting-edge medical techniques like gene treatments are paving the path for precision medicine by aiming to target particular genetic abnormalities linked to family forms of ALS. These treatments show promise for delaying or arresting the progression of disease by targeting the underlying genetic abnormalities. The path to regenerative therapies for ALS is paved with obstacles, though. Critical factors include ensuring the correct integration of donated cells, preventing harmful immunological reactions, and navigating the complex milieu of the deteriorating nervous system. Collaborative efforts between neurologists, geneticists, and regenerative medicine specialists are indispensable in advancing these therapies from experimental stages to viable clinical applications [39-41].

Schizophrenia

Schizophrenia's pathogenesis includes structural abnormalities in important brain regions, imbalances in neurotransmitters, and disruptions in neural connections. Regenerative therapies offer a fresh approach to treating this condition since they focus on restoring or replacing damaged brain circuits. By restoring structural integrity and functional balance inside the brain, neural stem cells and other regeneration techniques hope to reduce symptoms and improve cognitive function. Intriguing connections between neuroinflammation, oxidative stress, and the development of schizophrenia have been found by studies. Regenerative treatments that target these underlying causes have the potential to slow the progression of illness. By encouraging neurogenesis and synaptogenesis, it may be possible to harness the brain's capacity for regeneration, which could lead to a paradigm shift in how we treat schizophrenia.

However, the use of regenerative therapies in schizophrenia is still in its infancy and faces several difficulties. Precision medicine, which adapts regeneration techniques to the particular genetic and molecular profile of the individual, is essential. The difficulty of integrating these strategies into standard clinical practice is further highlighted by ethical considerations, safety issues, and the requirement for thorough clinical trials [42,43].

Autism Spectrum Disorder (ASD)

Atypical brain connections and synaptic function are the result of the neurodevelopmental causes of ASD, which entail complex interplay between genetic, environmental, and epigenetic variables. Regenerative therapies offer an appealing approach for addressing the underlying neurobiological disturbances in ASD because of their focus on restoring or altering brain circuits. In particular, stem cell therapies have a distinctive potential for regenerative interventions. It may be possible to encourage neurogenesis, repair damaged synaptic connections, and enhance the functionality of the entire brain network by taking advantage of the regenerative potential of neural stem cells. Although still in its infancy, the use of these therapies for ASD calls for careful evaluation of ethical, safety, and regulatory issues.

It is essential to note that while abnormal Nissl granule distribution is associated with these neurological disorders, the exact mechanisms linking Nissl granules to the disease pathologies may vary. The development of specific therapeutic treatments targeting Nissl granules themselves is challenging, given their fundamental role in protein synthesis and neuron function. However, understanding their involvement in these diseases can guide researchers in identifying potential therapeutic targets and developing strategies to address the underlying pathogenic processes that lead to abnormal Nissl granule distribution [44-47].

Neuroprotective strategies

To maintain neuronal structure and function, the major objective of neuroprotection is to avoid or minimize harm to the nervous system. Numerous strategies have been investigated, including pharmaceutical interventions, way-of-life changes, and cutting-edge technologies. The common mechanisms driving neurodegeneration, including oxidative stress, inflammation, and excitotoxicity, are routinely targeted by pharmacological neuroprotective treatments. These substances, which provide possible pathways for intervention, may include antioxidants, anti-inflammatory medications, and substances that affect neurotransmitter activity.

Neuroprotection is greatly aided by lifestyle changes like eating a balanced diet, getting frequent exercise, and engaging in mentally stimulating activities. In particular, physical activity has been linked to increased neuroplasticity, greater cognitive performance, and a decreased risk of neurodegenerative diseases. A balanced diet emphasizes consuming fruits, vegetables, whole grains, lean proteins, and healthy fats while minimizing the intake of processed foods, sugary beverages, and excessive amounts of saturated or trans fats. Key dietary components include antioxidants like vitamins C and E, omega-3 fatty acids, and polyphenols found in fruits, vegetables, and certain herbs. These compounds help combat oxidative stress and inflammation, which are underlying factors in many neurological disorders. New developments in genetic and neuroimaging studies offer insights into people's susceptibility to neurological illnesses in the field of emerging technologies. This individualized approach makes it easier to identify risk factors before they become problems, allowing for the introduction of focused neuroprotective measures. It is impossible to overestimate the value of prevention, and public health programs that raise risk factor knowledge and promote healthy lifestyle choices are essential for neuroprotection. Furthermore, finding new neuroprotective drugs and improving on current techniques depend on continual analysis and clinical studies [48-51].

Recent advances in cellular reprogramming and transdifferentiation

Cellular reprogramming essentially entails changing one type of cell into another, which is frequently accomplished by modifying gene expression patterns. Shinya Yamanaka's ground-breaking discovery of induced pluripotent stem cells (iPSCs) opened up previously unimaginable possibilities. iPSCs, created by reprogramming somatic cells, have characteristics similar to those of embryonic stem cells and provide a potentially endless source of patient-specific cells for regenerative applications.

Cellular reprogramming offers a paradigm shift in the treatment of degenerative illnesses in the clinical setting, shifting the emphasis from symptom control to tissue function recovery. Autologous cells can be created from patient-specific iPSCs for transplantation, reducing the possibility of immunological rejection. Despite the enormous promise of cellular reprogramming, difficulties still exist. Ongoing study and improvement are required because of the effectiveness and safety of reprogramming techniques, worries about tumorigenicity, and ethical problems. Additionally, a cautious approach is necessary to enable the responsible translation of cellular reprogramming technologies into clinical practice given the shifting regulatory environment [52-54].

Transdifferentiation is fundamentally the process of changing one kind of specialized cell into another without returning to a pluripotent or stem cell state. This approach puts to rest the conventional view of cellular fate and creates fresh opportunities for utilizing the body's capacity for regeneration. Transdifferentiation enables a more direct and targeted cellular conversion than iPSCs, which require reprogramming cells to a pluripotent state before redifferentiation.

Transdifferentiation offers possible treatments for congenital abnormalities, degenerative diseases, and traumas in the therapeutic setting. To induce targeted transdifferentiation for therapeutic purposes, researchers can develop techniques by studying the molecular cues that cause cell fate alterations. Optimizing effectiveness, maintaining functional integration of transformed cells, and resolving safety issues such as tumorigenicity are challenges related to transdifferentiation. Transdifferentiation procedures are being improved through ongoing research to make them more dependable and useful in a variety of clinical settings [55-57].

## Conclusions

A fascinating area in our search for novel medical treatments is the complex connection between Nissl granules, axonal regeneration, and the larger field of regenerative therapeutics. Nissl granules, which were long primarily understood for their function in the manufacture of neuronal proteins, are now recognized as dynamic components of the nervous system's regenerative processes. At the intersection of cellular resilience and recovery, the link between Nissl granules and axonal regeneration takes shape. These granules, which are found in the soma of neurons, are crucial to the regeneration of injured axons and are an integral part of the complex process of brain healing. A promising approach to utilizing the inherent regenerative capacity of our neurons is to comprehend the complex chemical mechanisms governing this interaction.

In the broader context of regenerative treatments, the investigation of Nissl granules provides doors for focused interventions that go beyond symptomatic relief. Understanding the intricate workings of how these granules affect axonal regeneration reveals possibilities for enhancing the nervous system's inherent repair mechanisms. The repair of functional neuronal connections is crucial in the treatment of neurological illnesses, neurodegenerative diseases, and traumatic injuries. We set out on a road that connects molecular findings with game-changing clinical applications by embracing the potential synergy between Nissl granules, axonal regeneration, and regenerative therapies. As healthcare providers, our shared commitment to comprehending and utilizing these complex biological processes offers the potential to reshape the field of neurological care and give patients who are seeking more than simply management hope for a meaningful restoration of neural function and quality of life.
